# MiR-92 suppresses proliferation and induces apoptosis by targeting EP4/Notch1 axis in gastric cancer

**DOI:** 10.18632/oncotarget.24819

**Published:** 2018-05-11

**Authors:** Vivian Yvonne Shin, Man-Ting Siu, Xin Liu, Enders K.O. Ng, Ava Kwong, Kent-Man Chu

**Affiliations:** ^1^ Department of Surgery, The University of Hong Kong, Hong Kong SAR; ^2^ Department of Surgery, Hong Kong Sanatorium and Hospital, Hong Kong SAR; ^3^ Hong Kong Hereditary Breast Cancer Family Registry, Hong Kong SAR

**Keywords:** miR-92, NF-κB, EP4, Notch1, gastric cancer

## Abstract

MiR-92a has been shown to be dysregulated in various cancers and exhibited differential role in carcinogenesis. In this study, we sought to delineate the functional role of miR-92a and its regulatory pathway in gastric cancer. MiR-92a expression were underexpressed in tissues of gastric cancer patients with the area under curve (AUC) of 0.78. Low expression in plasma was due to the increased promoter DNA methylation of miR-92a. Overexpression of miR-92a inhibited cell proliferation and invasion, and induced apoptosis. Furthermore, miR-92a reduced tumor growth in xenograft model. EP4 and Notch 1 were identified to be negatively regulated by miR-92a, and involved in cell growth. Moreover, NF-κB expression was inversely correlated with miR-92a in gastric cancer tissues and suppressed the expression of miR-92. This study unravels the tumor suppressive role of miR-92a involving EP4/Notch 1 signaling regulated by NF-κB in gastric cancer. Further studies on miR-92a and EP4/Notch1 may provide a new treatment strategy for gastric cancer.

## INTRODUCTION

Gastric cancer is the second leading cause of cancer mortality in the world, and over 952,000 cases newly diagnosed and 723,000 deaths in 2012 [1]. The incidence of gastric cancer is particularly high in Asia, including China, Korea and Japan. Surgery remains the keystone of curative treatment of gastric cancer, however, most cases are diagnosed at advanced stage due to late presentation. The 5-year overall survival rate for all patients with gastric cancer is about 28% in the US [1]. Thus, early diagnosis and development of new drugs may help to reduce the overall mortality.

MicroRNAs (miRNAs) are endogenous noncoding regulatory RNAs with 17–25 nucleotides that involves in post-transcriptional gene regulation. The ability to bind complementary sequences in 3′-untranslated regions (3′-UTR) of various target mRNAs leading to direct mRNA degradation or translational repression. Emerging evidence suggested that microRNAs (miRNAs) contributed to various human malignancies, including gastric cancer [2, 3]. Accumulating evidence showed that the miRNA expression profiles were different in cancerous tissues and normal counterparts [6, 7]. The fact that miRNAs are very specific for different types of tissues and even for types of cells within those tissues, many researchers are urged to profile the miRNA patterns in various cancer types, which put forward the diagnostic and prognostic values of miRNA in clinical applications. miRNAs regulate gene expression and contribute to development, differentiation, inflammation, and carcinogenesis [4, 5]. They may function as tumor suppressors or oncogenes during the process of cancer development [8].

A polycistronic miR-17-92 cluster contain 6 mature miRs (miR-17, miR-18a, miR-19a, miR-19b, miR-20a and miR-92a), and is located on human chromosome 13q31. Overexpression of miR-17-92 cluster contributes to the development of B-cell lymphoma and other cancers [9, 10]. These miRNAs are believed to involve in the various cellular events in different organs, however, the detailed mechanism has not been fully studied. In patients with esophageal squamous cell carcinoma (ESCC), the level of miR-92a was highly expressed in tumor tissues than adjacent non-tumor counterparts [11]. The expression level was directly correlated with the status of lymph node metastasis and TNM stage. The oncogenic property of miR-92a was also seen in the plasma of patients with colorectal cancer [12]. Conversely, the levels of miR-92a were markedly reduced in acute leukemia and breast cancer patients [13, 14]. Whether miR-92a exhibits oncogenic or tumor suppressive property in gastric carcinogenesis is controversial, and needs further study.

In this present study, we profiled the miRNA expression in plasma of gastric cancer patients using array platform, and was further validated in plasma and cancerous tissues. We showed that the downregulation of miR-92a occurred frequently in gastric tumor tissues and cancer cell lines. Ectopic expression of miR-92a markedly retarded gastric cancer cell growth and invasion. Inhibition of miR-92a suppressed apoptosis by decreasing caspase-3 and PARP expressions. Moreover, we identified miR-92a is negatively regulated by NF-κB through EP4/Notch 1 signaling pathway. These findings delineate an important tumor suppressive property of miR-92a in the control of gastric cancer cell proliferation.

## RESULTS

### Expression of miR-92a is frequently downregulated in human gastric tissues

**Figure 1:**
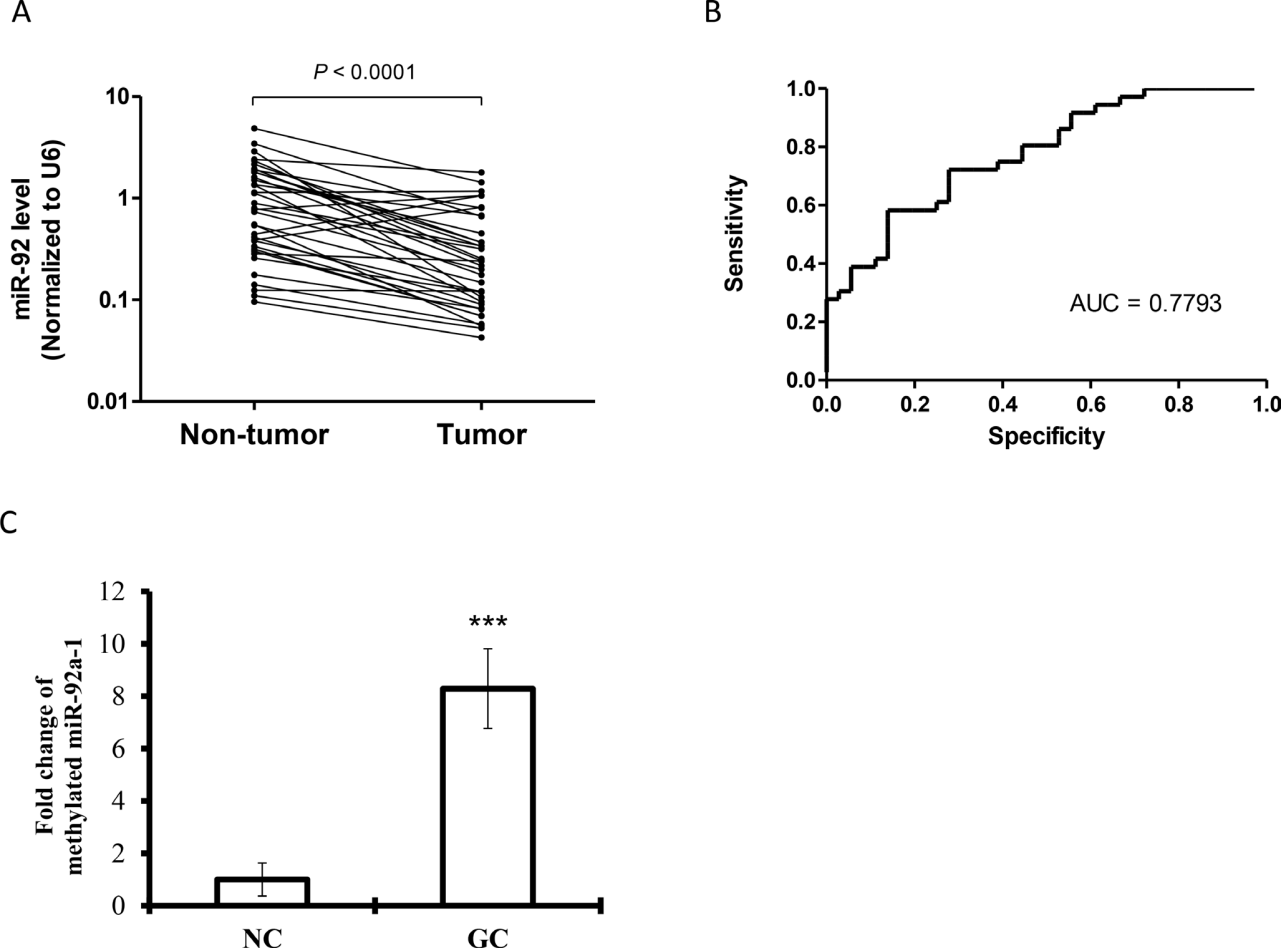
Expression levels of (**A**) miR-92a in paired gastric tumor tissues and adjacent non-tumor counterparts (*n* = 36). Expression levels were normalized to U6B. Statistically significant differences were analyzed using Wilcoxon test. (**B**) Receiver-operating characteristic (ROC) curve analysis of miRNA for discriminating gastric cancer patients from healthy controls. (**C**) Methylated miR-92a-1 DNA expression in plasma samples from gastric cancer patients and healthy controls by MSRED-qPCR. ^***^P < 0.001 is considered as statistically significance.

Previous results from the microarray data revealed that 82 miRNAs were significantly differentiated between gastric cancer cases and normal control [15]. MiR-92a was found to be significantly downregulated in the plasma of gastric cancer patients. We then further validated the expression of miR-92a in tissue samples by independent quantitative RT-PCR. The underexpressed miR-92a was seen in a subset of 36 paired gastric cancer samples, comparing tumor tissues against adjacent non-tumor counterparts (Figure 1A) with the area under curve (AUC) of 0.78 (Figure 1B). Promoter hypermethylation of miR-92a was examined by MSRED-qPCR, plasma DNA in gastric cancer patients showed a higher methylation level (Figure 1C), this partly explain the low expression of miR-92a in gastric cancer.

### Tumor suppressive role of miR-92a in gastric cancer *in vitro* and *in vivo*

**Figure 2:**
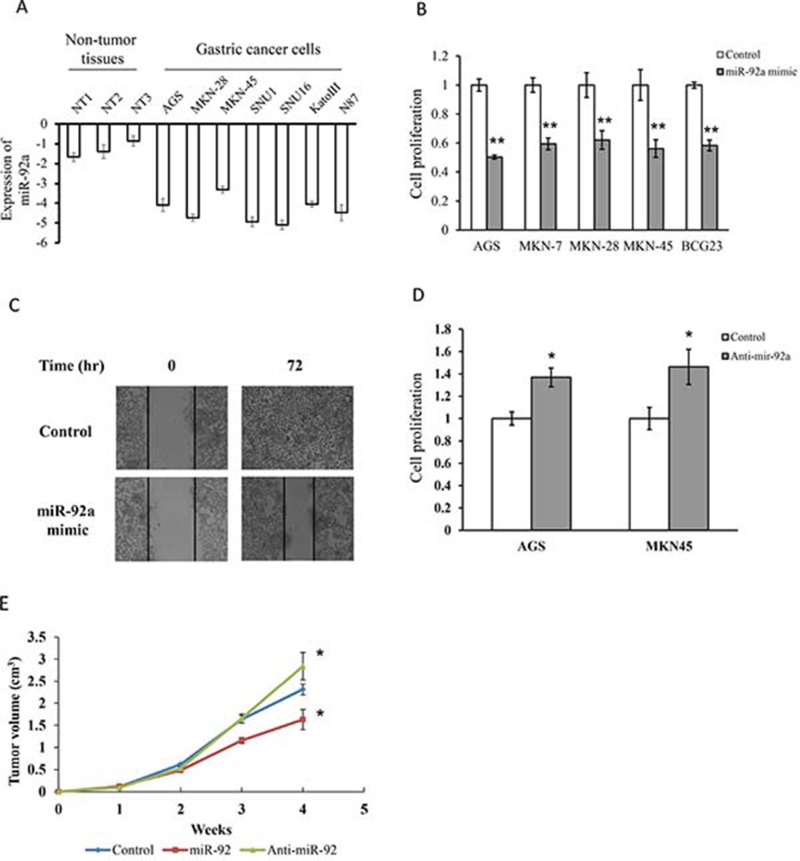
MiR-92a suppressed cell proliferation and migration in gastric cancer cells (**A**) Expression of miR-92a in normal gastric tissues and gastric cancer cells. (**B**) Transfection of cells with miR-92a mimics in five gastric cancer cells for 48 hr and determine cell proliferation by MTT assay. (**C**) Cells were transfected with miR-92a mimics and determine cell migration in MKN-45 by scratch wound healing assay. (**D**) Cell proliferation was evaluated in cells transfected with anit-miR-92a in AGS and MKN-45. (**E**) Tumor suppressive property of miR-92a was assessed in xenograft model. MKN-45 cells were injected into the flank of nude mice, and was sacrificed after 4 weeks. ^*^P < 0.05 and ^**^P < 0.01 are considered as statistically significance.

The downregulation of miR-92a in gastric cancer may implicate a potential tumor suppressive role in gastric tumor. All gastric cancer cells had a very low expression of miR-92a when compared to human non-tumor tissues (Figure 2A). To test this hypothesis, we examined the functional role of miR-92a on cell viability, we transfected the cells with miR-92a mimic in five gastric cancer cell lines (AGS, MKN-7, MKN-28, MKN-45 and NCI-N87). Ectopic expression of miR-92a markedly induced growth retardation by more than 30% in all cell lines (Figure 2B). In particular, miR-92a reduced cell viability by 40% and 42% in AGS and MKN-45 respectively. Moreover, cell migration was also retarded by miR-92a overexpression in AGS (Figure 2C). On the other hand, transfection with anti-miR-92a stimulated cell proliferation when compared with the control transfected cells, implicating the growth inhibitory effect of miR-92a in gastric cancer (Figure 2D).

We further examined whether miR-92a will retard tumor growth in human xenograft model. MiR-92a, anti-miR-92a and negative control transfected cells were implanted subcutaneously on the right flank of the mice and tumor volumes were compared at week 4. As shown in Figure 2E, tumor volume rapidly increased from 2 weeks in all groups and miR-92a transfected mice had a smaller tumor volume than control mice. Moreover, the tumor volume of anti-miR-92a transfected mice was markedly increased when compared with the control mice. These data suggested that miR-92a exhibited anti-tumorigenic property both *in vitro* and *in vivo*.

### Overexpression of miR-92a induced cell apoptosis

**Figure 3:**
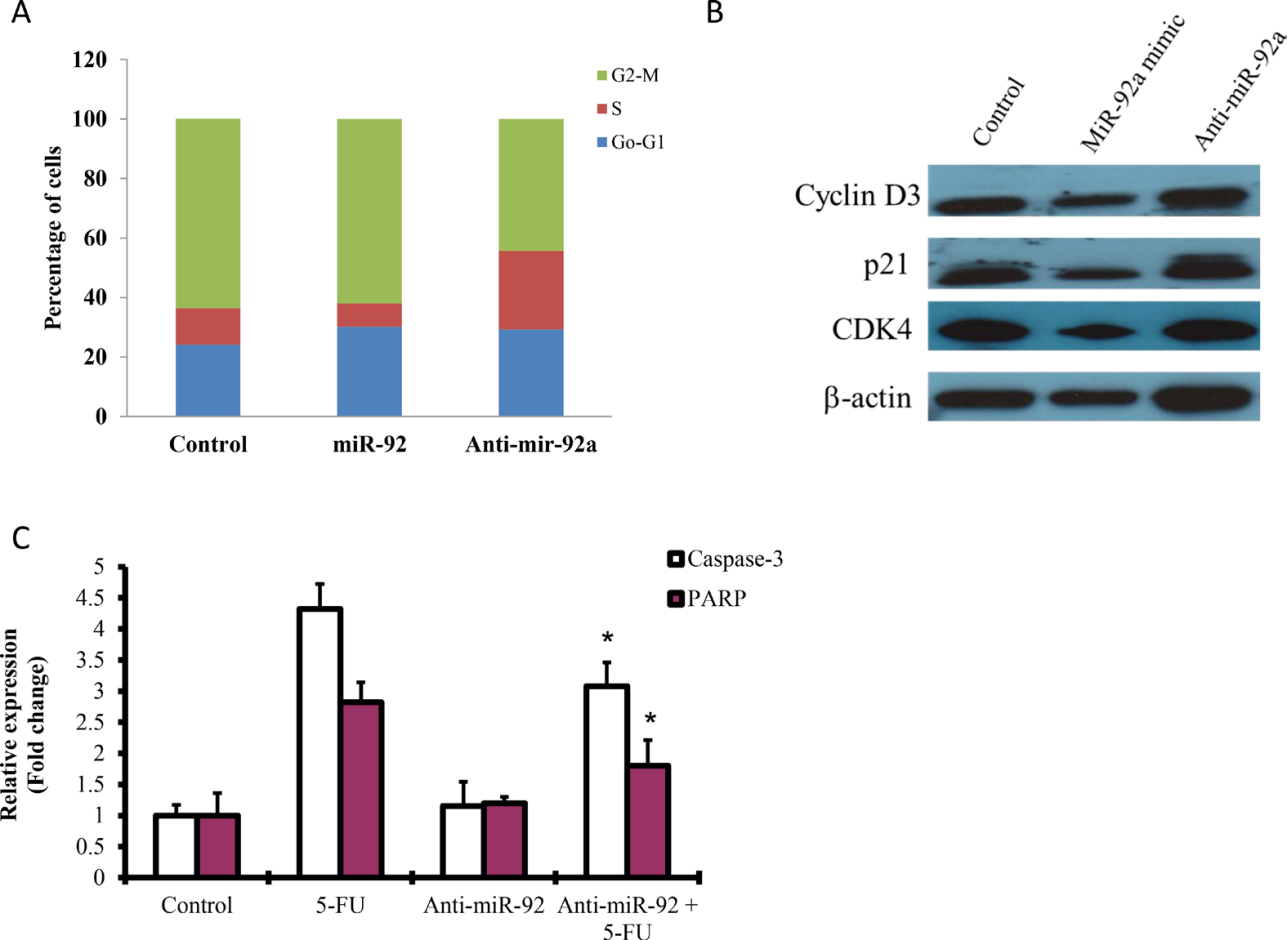
Effect of miR-92a on cell cycle analysis and apoptosis (**A**) MKN-45 cells were transfected with miR-92a mimic and anti-miR-92a for 48 hr and subjected to flow cytometry analysis. (**B**) Western blot analysis of cyclin D3, p21 and CDK4 in cells transfected with miR-92a mimic and anti-miR-92a. (**C**) Cells transfected with anti-miR-92a on apoptosis was measured by ELISA. 5-Fluorouracil (5-FU) was used as a positive control to induce apoptosis. ^*^P < 0.05 is considered as statistically significance.

To examine the tumor suppressive properties of miR-92a on gastric cancer cell growth, we performed cell cycle analysis and apoptosis in MKN-45 cells. Results showed that miR-92a transfected cells appeared to have lower percentage of S phase cells compared to control (Figure 3A). On the other hand, anti-miR-92a transfected cells had a significant higher percentage of S phase cells, and caused a reduction in G2/M phase. Western blot analysis showed that cyclin D3 and cyclin dependent kinase 4 (CDK4) were reduced by miR-92a (Figure 3B).

Apoptosis has been implicated in carcinogenesis, we investigated whether miR-92a would have any effect on cell death. Cells treated with 5-fluorouracil (5-FU) markedly induced caspase-3 and PARP, as detected by ELISA. Transfection with anit-miR-92a significantly blocked 5-FU-induced apoptosis by reducing caspase-3 and PARP expressions (Figure 3C).

### MiR-92 is a negative regulator of Notch and EP4 signaling

**Figure 4:**
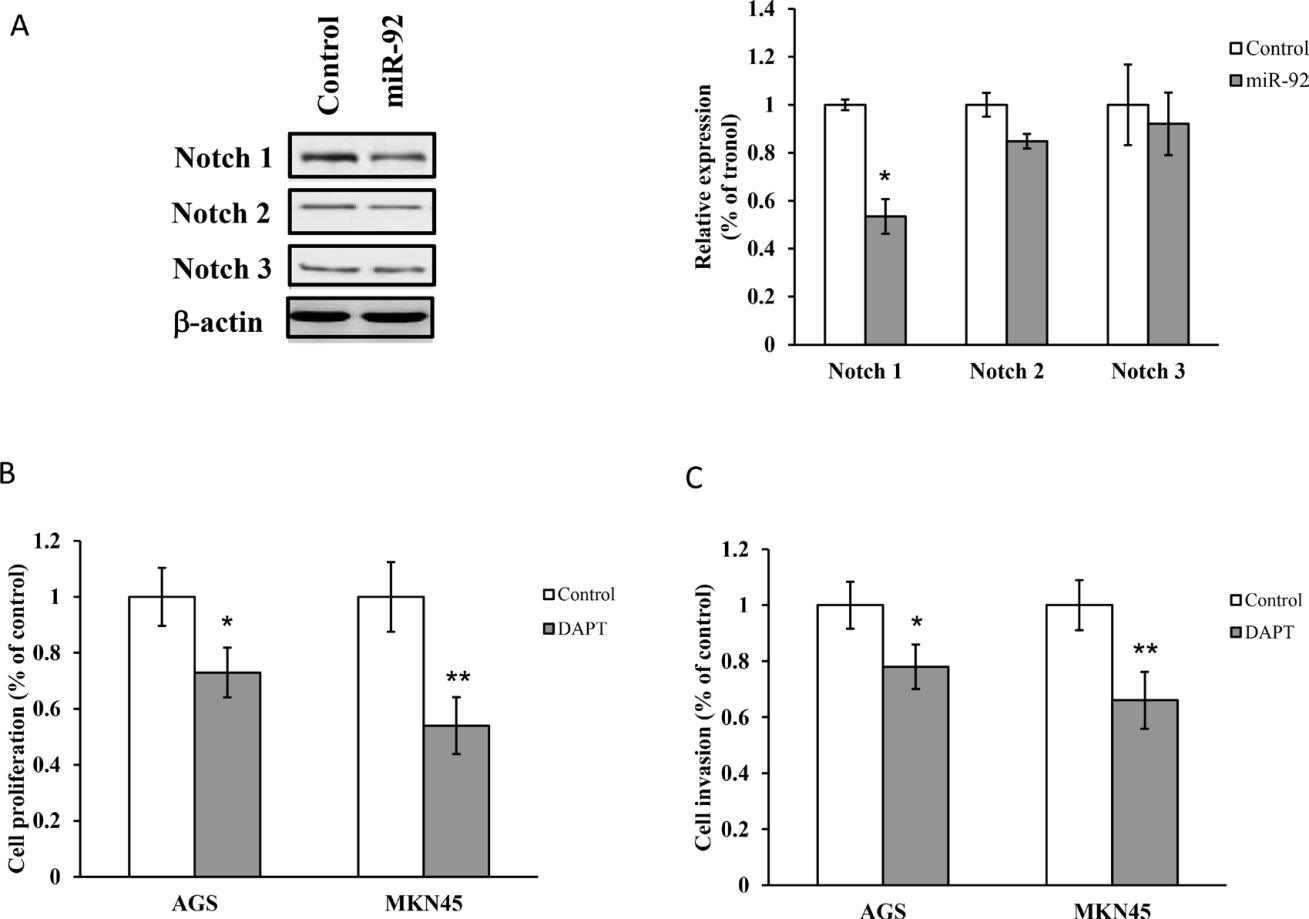
Notch is regulated by miR-92a (**A**) Western blot analysis of Notch 1, Notch 2 and Notch 3 in cells transfected with miR-92a mimic. (**B**) Cell proliferation and (**C**) invasion were evaluated in cells treated with DAPT (γ-secretase inhibitor, 5 μM) by MTT assay and Matrigel Invasion Chamber respectively. ^*^P < 0.05 and ^**^P < 0.01 are considered as statistically significance.

Activation of Notch signaling has been evidenced in gastric cancer growth and had high expression level in human gastric cancer tissues [16]. Expressions of Notch 1, Notch 2 and Notch 3 were constitutively expressed in MKN-45 cells, while ectopic expression of miR-92a significantly reduced expression of Notch 1, but not Notch 2 and Notch 3 (Figure 4A). Inhibition of Notch 1 by N-[N-(3, 5-difluorophenacetyl)-L-alanyl]-S-phenylglycine t-butyl ester (DAPT, γ-secretase inhibitor, 5 μM) significantly suppressed cell proliferation and cell migration (Figure 4B and 4C).

**Figure 5:**
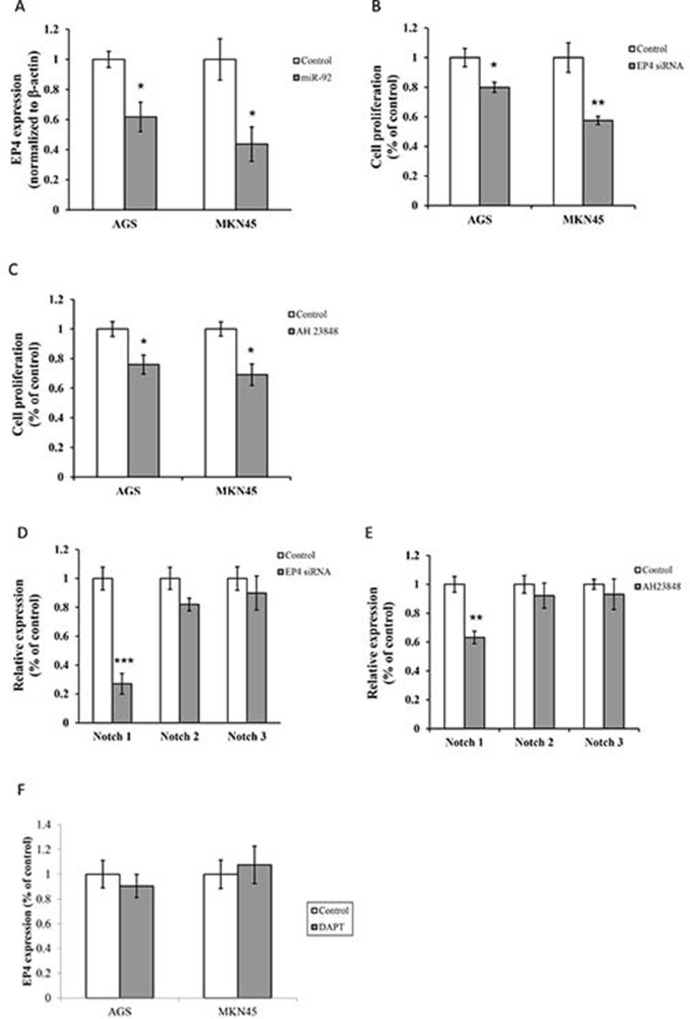
MiR-92 is a negative regulator of Notch and EP4 signaling (**A**) Expression of EP4 were detected by real-time RT-PCR. Cell proliferation was assessed in cells treated with (**B**) EP4 siRNA and (**C**) AH 23848 (EP4 antagonist, 10 μM). Expressions of Notch 1, Notch 2 and Notch 3 were analyzed in cells treated with (**D**) EP4 siRNA and (**E**) AH 23848 (10 μM) by real-time RT-PCR. (**F**) Expression of EP4 in cells treated with DAPT (5 μM) was measured by real-time RT-PCR. ^*^P < 0.05, ^**^P < 0.01 and ^***^P < 0.001 are considered as statistically significance.

On the other hand, miR-92a overexpression reduced EP4 receptor expression (Figure 5A). Treatment with EP4 siRNA or EP4 antagonist (AH 23848, 10 μM) markedly retarded cell proliferation and lowered the expression of Notch1 (Figure 5B–5E). On contrary, inhibition of Notch 1 by DAPT (5 μM) suppressed cell proliferation, but not the expression of EP4 receptor (Figure 5F). Moreover, blockade of EP4 and Notch 1 did not alter the expression of miR-92a (data not shown), implicating that miR-92a regulated cancer cell growth through EP4/Notch 1 signaling pathway.

### NF-κB regulated miR-92a expression

**Figure 6:**
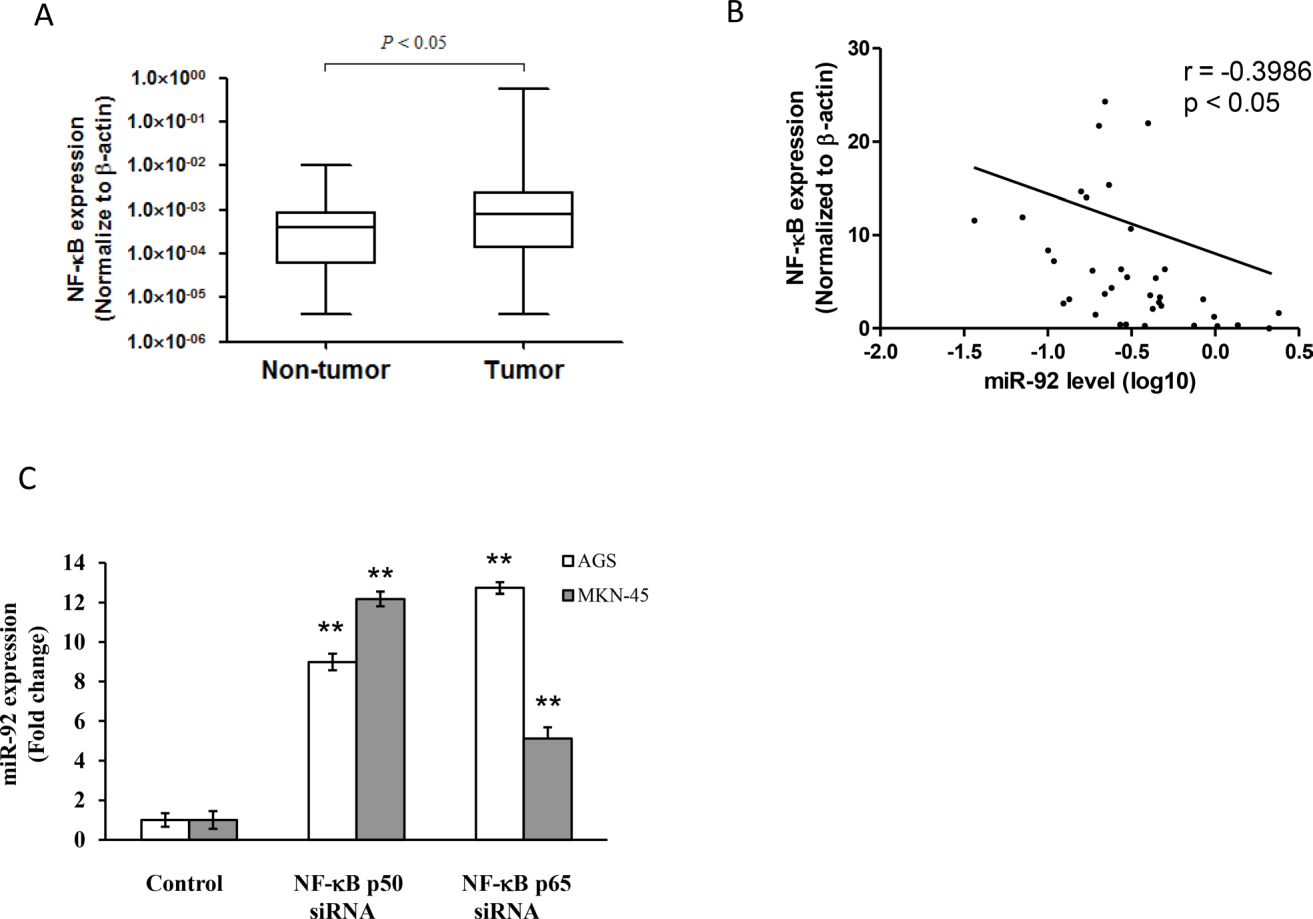
NF-κB regulated miR-92 expression in gastric cancer (**A**) Expression levels were normalized to β-actin. Box plot of NF-κB expression in primary tissues of gastric cancer patients (*n* = 36). The boxes mark the interval between the 25th and 75th percentiles, and the lines inside the box denote the medians. The whiskers represent the interval between the 10th and 90th percentiles. Statistically significant differences were analyzed using Mann–Whitney test. (**B**) Negative correlation between NF-κB expression and miR-92a levels in primary tissues of gastric cancer patients. (**C**) Expression of miR-92a in cells transfected with NF-κB p50 and p65 siRNA were measured by real-time RT-PCR. ^**^P < 0.01 is considered as statistically significance.

NF-κB expression was higher in primary tumors than paired non-tumor tissues (Figure 6A). We found that NF-κB expression is negatively regulated with miR-92 level in gastric tissues (Figure 6B). Transfection with NF-κB siRNA (p50 and p65) increased miR-92 expression in gastric cancer (Figure 6C).

## DISCUSSION

Dysregulation of miRNA has been the hallmark of human malignancies, including gastric cancer. Many studies have been profile the miRNA signature for specific cancer, however, the functional roles of individual miRNA on gastric cancer remain elusive. The expression of individual miRNA may vary in different tissues and the origin of the tumor. MiR-92a is transcribed from miR-17-92 locus, which was first discovered to be oncogenic in B-cell lymphoma and lung cancer [9]. It is located within ~1 kb of an intron of the C13-25orf locus at 13q31-q32 where this region is frequently amplified and overexpressed in lymphoma [17]. MiR-92a has been shown to be upregulated in solid tumors, including colon, esophagus, prostate and stomach [7, 11, 18]. Overexpression of miR-92 has been observed in colorectal cancer tissues when compared with adjacent counterparts [19], as well as in plasma of colorectal cancer patients [12]. Similar observation was also seen in hepatocellular cancerous tissues and cell lines [20]. Ectopic expression of miR-92a promoted cell growth in different hepatoma cell lines, while anti-miR-92a significantly retarded the growth. Interestingly, the expression of miR-92a was lowered in the plasma of hepatocellular carcinoma patients, while it was increased after tumor resection. The author suggested that low expression of miR-92a can serve as a diagnostic marker in plasma of hepatocellular carcinoma and leukemia patients [13]. Currently, the low expression of miR-92a was found to be associated with tumor stage and disease-free survival in breast cancer patients. The role of miR-92a on tumor-stromal interactions was evidenced by macrophage infiltration, which might involve in tumor development and progression. This controversy prompts us to explore the potential role of miR-92a in gastric cancer. In this report, we validated the expression of miR-92a in primary tumors of gastric cancer patients by real-time PCR. Several proofs in this study demonstrated that restoration of miR-92a inhibited cell proliferation and cell invasion, and induced apoptosis in MKN-45 and AGS cells. Moreover, low expression of miR-92a in gastric cancer was due to epigenetic deregulation as demonstrated by MSRED-qPCR.

Notch signaling has been implicated in various carcinogenesis, including gastric cancer. Phosphorylation of STAT3 and Twist promoted gastric cancer progression were regulated by Notch 1 receptor intracellular domain [21]. Expressions of Notch 1 and Jagged1 were positively correlated with p-STAT3 levels in primary tumor tissues. Notch 2 receptor intracellular domain regulated COX-2 to promote epithelial-mesenchymal transition and cell invasion [22]. Several studies have shown that Notch 1 and Notch 2 expressions were detected in cancerous tissues when compared with normal mucosa, and they were correlated with gastric cancer formation [23]. We found that Notch 1 was highly expressed in gastric cancer tissues than normal counterparts by real-time RT-PCR. Inhibition of Notch 1 by DAPT (inhibitor of γ-secretase) significantly blocked cell invasion and growth in gastric cancer.

Prostaglandin E_2_ (PGE_2_) plays significant role in cancer initiation and progression through its receptors (EP receptor). There are four G-protein-coupled cell surface receptors subtypes of PGE receptors, namely EP1, EP2, EP3 and EP4, for signal transduction. These receptors are coupled to different signaling pathways and cell functions. There is strong evidence showing that PGE_2_ and its receptors were implicated in carcinogenesis of different types of tumors, including gastric cancer. We found that EP2 and EP4 receptors are involved in cigarette-induced gastric cancer growth. Knockdown of EP2 or EP4 suppressed tumor growth, angiogenesis and induced apoptosis through p38 phosphorylation [24]. In another study, we demonstrated that miR-16 and miR-21 expression were upregulated by nicotine, silencing of miR-16/21 markedly reduced cell proliferation [25]. Transfection with EP2 or EP4 siRNA abrogated the expressions of miR-16/21, and resulted in growth retardation. Several studies reported that activation of EP4 receptor would promote healing of bone fracture [26] and wound in the stomach and intestine [27, 28]. The anti-tumorigenic effect of EP4 antagonist was evidenced by enhanced apoptosis and reduced VEGF proteins in breast cancer [29]. In this study, we demonstrated silencing of EP4 receptor by siRNA or antagonist caused significant growth retardation in gastric cancer cell lines. Restoration of miR-92a expression suppressed cell proliferation and induced apoptosis through the downregulation of EP4 receptor. It is therefore suggested that miR-92a regulated cell proliferation and cell invasion through EP4/Notch 1 signaling pathway.

**Figure 7:**
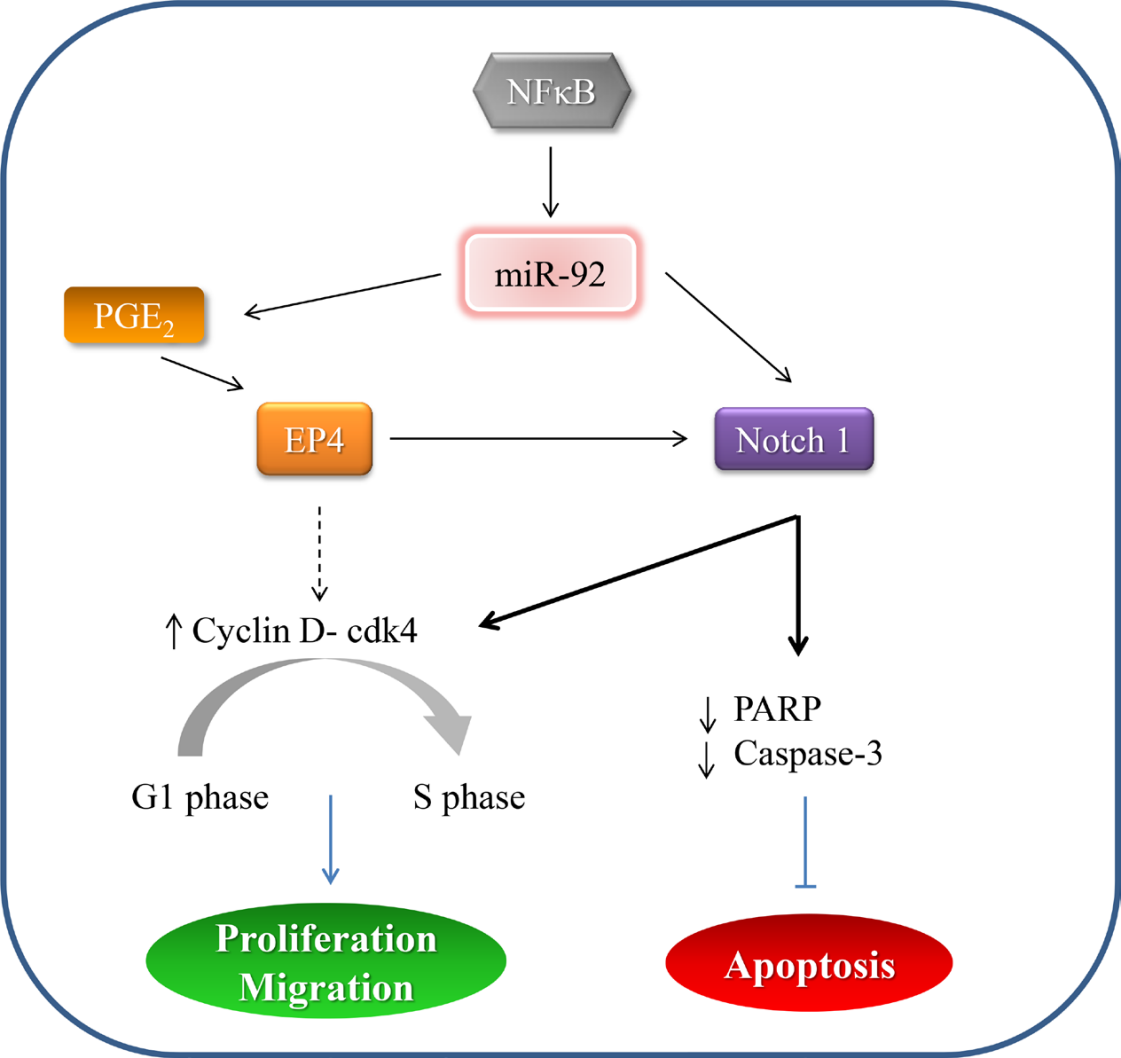
A proposed mechanism depicting miR-92a signaling in gastric cancer

Emerging evidence reported that important roles of miRNA in pathogenesis of various human malignancies by regulating gene expressions. It is believed that a single miRNA would regulate several mRNAs in different signaling cascades, hence manipulation of one miRNA would be more effective in the therapeutic perspective. In this study, we have shown that miR-92a is deregulated in primary tissues of gastric cancer patients. Perhaps this is the first report unravel the tumor suppressive property of miR-92a in gastric cancer, and the downstream signaling pathway involving EP4/Notch 1 signaling regulated by NF-κB. A proposed mechanism has been illustrated in Figure 7. Further study is warranted to evaluate the feasibility of using miR-92a as anti-cancer therapy in gastric cancer patients.

## MATERIALS AND METHODS

### Reagents and drugs

Antibodies specific to NF-κB p50 and p65 were purchased from Santa Cruz Biotechnology (Santa Cruz, CA, USA). Other chemicals and reagents were from Sigma (St Louis, MO, USA) unless otherwise specified.

### Clinical samples

Plasma and tissue samples are stored at the frozen tissue bank of the Department. The collection and storage of such tissue samples have been approved by the Institutional Review Board. Informed consent has been obtained from each patient. Consecutive patients (*n* = 36) with confirmed diagnosis of gastric adenocarcinoma who undergo surgical resection will be included. Classification and staging of tumour will be based on the Lauren's classification and both the Japanese and UICC staging classification, respectively. Characteristics of patients such as gender, age, co-morbidity, presenting symptoms and signs, laboratory investigation, operative findings, and type of resection will be prospectively collected into our standard electronic database. Clinical characteristics of gastric cancer patients were summarized in Table 1.

**Table 1:** Clinical characteristics of gastric cancer patients

	No. of cases (*n* = 36)	Total
Age [years; mean (SD)]	65.6 (16.8)	
Sex		
Men	24	24/36 (66.67%)
Women	12	12/36 (33.33%)
Depth of Invasion (T)		
T1	5	5/36 (13.89%)
T2	11	11/36 (30.56%)
T3	17	17/36 (47.22%)
T4	3	3/36 (8.33%)
Lymph-node metastasis (N)		
N0	6	6/36 (16.67%)
N1	8	8/36 (22.22%)
N2	21	21/36 (58.33%)
N3	1	1/36 (2.78%)
Distant metastasis		
No	27	27/36 (75%)
Yes	9	9/36 (25%)
Stage		
I	4	4/36 (11.11%)
II	10	10/36 (27.78%)
III	12	12/36 (33.33%)
IV	10	10/36 (27.78%)

### Plasma DNA extraction and MSRED-qPCR

Plasma DNAs were extracted using QIAmp DNA Blood Mini kit (Qiagen), as previously described with modifications [30]. In brief, 600 μl of plasma was used and DNA was eluted with 40 μl of DNase-free water. 30 μl of extracted plasma DNA was digested in a 40 μl reaction volume with 30 U of methylation-sensitive restriction enzyme, BstU1 (New England BioLabs), at 60°C for 16 hr. To ensure complete enzyme digestion, it was run in parallel with a positive and a negative control digestion in which 30 ng of completely methylated or unmethylated control DNA (EpiTect Control DNA, Qiagen) was digested. After digestion, same amount of digested or undigested plasma DNA along with control digestion was subjected to qPCR using QuantiTect SYBR Green PCR Kit (Qiagen) in ABI 7900 HT system (Applied Biosystems). Each reaction is performed in a final volume of 20 μl containing digested (1.3 μl) or undigested (1 μl) plasma DNA, 500 nM each primer and 1× SYBR Green PCR Master mix (Qiagen). At the end of the PCR cycles, melting curve analyses are performed to validate the specific PCR product. Primers used for this qPCR were: Forward, 5′-CCGCGTGCTGGGATTC and Reverse, 5′-TCCAGAAGGCTGCAAATGG. Relative expression level of plasma methylated miR-92 was expressed as 2^ΔCt(undigest-digest)^. ΔCt_(undigest-digest)_ was calculated by subtracting the Ct values of digested plasma DNA from the Ct values of undigested plasma DNA. Since Ct of undigest should be ≤Ct of digest, the expression level ranges from 1 to 0. Each sample is run in duplicates for analysis. For 100% digestion efficiency, relative expression level of completely unmethylated control DNA (2^ΔCt(CTRLundig-CTRLdig)^) must be close to zero whereas the level of completely methylated control must be 1.

### Cell culture

Gastric cell lines AGS and MKN-45, obtained from American Type Culture Collection (ATCC, Rockville, MD, USA) and Riken Cell Bank (Tsukuba, Japan) were cultured in RMPI 1640 medium (GibcoBRL, Grand Island, USA) supplemented with 10% fetal bovine serum in humid atmosphere containing 5% carbon dioxide.

### SiRNA meditated gene knock-down

Cells were grown in antibiotic-free medium and transfected with siRNAs (Qiagen, Hilden, Germany) for validated human EP4, NF-κB p50 and p65 using Lipofectamine 2000 (Invitrogen, Carlsbad, CA, USA). For miRNA transfection, we incubated synthetic miRNA mimic or inhibitor (Qiagen)with HiPerFect transfection reagent. RNA and protein were extracted after transfection for 48 hr.

### MTT assay

Cells were seeded in 96-well plate and treated with miR-92 mimic or control oligo (Qiagen). After 48 hr, the culture medium was discarded and restained with 3-(4,5-Dimethyl-2-thiazolyl)-2,5-diphenyl-2H-tetrazolium bromide (MTT) (5 mg/ml) for 3 hr. The absorbance was measured at 570 nm on a microplate reader.

### Invasion assay

Cell invasiveness was measured by using 24-well transwell plate BioCoat Matrigel Invasion Chamber (BD Biosciences) as described previously [31]. Briefly, cells were transfected with miR-92 mimic or control oligo for 48 hr and re-suspended 4 × 10^4^ cells in medium without serum or growth factors in the upper chamber. The lower chamber was filled with culture medium supplemented with 10% FBS as chemoattractant. After 24 hr, the invaded cells that had passed through the Matrigel were fixed in 100% methanol, stained with 0.1% crystal violet and counted under a light microscope (x200).

### Scratch wound healing assay

Cells were seeded on 24-well plate until confluent, and gently scratch a straight line with a 1 ml pipette tip across the well. After washing, cells were then treated with miR-92 mimic or control oligo for 48 hr. Images of the wound healing were taken at different time points and were captured by DP controller 3.31.292 (Olympus, MA, USA).

### Apoptosis

Cell lysates were used to detect apoptosis using a commercially available ELISA kit from Cell Signaling (PathScan^®^ Apoptosis Multi-target Sandwich ELISA kit) according to the manufacturer's protocol. Briefly, cells were transfected with miR-92 mimic or control oligo for 48 hr in a 24-well plate. After transfection, cell lysates were sonicated and centrifuged for 10 min at 4°C. Dilute the cell lysates with sample diluent and incubated overnight at 4°C. Afterwards, incubate antibodies (phospho-p53, cleaved caspase-3, PARP and phospho-Bad) for 1 hr at 37°C, followed by HRP-linked secondary antibody and incubated for another 30 min at 37°C. TMB substrate and stop solution were added and measured the absorbance at 450 nm.

### Tumorigenicity

Human xenograft models were used to study the tumor suppressive property of miR-92a. This study was approved by the Committee on the Use of Live Animals in Teaching and Research (CULATR) in the University of Hong Kong. MB-231 cells were trypsinized and the final cell concentration was adjusted to 1 × 10^6^ cells/ml. One hundred microliters of cell suspension were injected into the flank of nude mice. Xenografted tumor size was assessed by external caliper weekly and was calculated by using formula V = L × W^2^/2 where V = tumor volume, L = tumor length and W = tumor width [32]. Mice were sacrificed after 4 weeks and inoculated tumors were extracted for histological examination.

### Real-time reverse transcription-polymerase chain reaction

Total RNA was extracted from tissues with Tri-Reagent according to the protocol provided. Extracted RNA was purified by genomic DNA elimination mixture, and reversed-transcribed into cDNA using miScript Reverse Transcription kit (Qiagen). Real-time PCR was performed using Power SYBR-Green real-time PCR system and ABI PRISM 7500 detection system (Applied Biosystems). The miR-92a forward primer sequence was 5′-TATTGCACTTGTCCCGGCCTGT-3′ (MIMAT0000092, from miRBase). U6B was used for normalization. The reaction conditions were 60°C for 5 min, 95°C for 10 min, 95°C for 15 sec and 60°C for 1 min (40 cycles). Fold change in expression of each gene is calculated by a comparative threshold cycle (C_t_) method using the formula: 2^-[DCt(experiment)- DCt(control)]^.

### SDS-PAGE and Western blot analysis

Cellular proteins were extracted and resolved in SDS-PAGE gel and followed the standard procedures. Briefly, proteins were transferred to a Hybond C nitrocellulose membrane (Amersham). The membrane was blocked with 5% non-fat milk probed with indicated antibodies. Membranes were developed by the enhanced chemiluminescence system (Amersham) and exposed to an X-ray film (Fuji Photo Film Co. Ltd., Tokyo, Japan). Quantification was carried out by a video densitometer (Scan Maker III, Microtek, USA). The relative intensities of the proteins were normalized to respective β-actin control.

### Statistical analysis

Results are expressed as mean ± SD. Statistical analysis was performed with two-tailed Student's *t*-test to compare data between two groups, and one-way ANOVA with Tukey post hoc was applied to compare three or more groups. *P* values < 0.05 were considered statistically significant

## References

[1] Hundahl SA, Phillips JL, Menck HR (2000). The National Cancer Data Base Report on poor survival of U.S. gastric carcinoma patients treated with gastrectomy: Fifth Edition American Joint Committee on Cancer staging, proximal disease, and the “different disease” hypothesis. Cancer.

[2] Ueda T, Volinia S, Okumura H, Shimizu M, Taccioli C, Rossi S, Alder H, Liu CG, Oue N, Yasui W, Yoshida K, Sasaki H, Nomura S (2010). Relation between microRNA expression and progression and prognosis of gastric cancer: a microRNA expression analysis. Lancet Oncol.

[3] Ribeiro-dos-Santos A, Khayat AS, Silva A, Alencar DO, Lobato J, Luz L, Pinheiro DG, Varuzza L, Assumpcao M, Assumpcao P, Santos S, Zanette DL, Silva WA (2010). Ultra-deep sequencing reveals the microRNA expression pattern of the human stomach. PLoS One.

[4] He L, Hannon GJ (2004). MicroRNAs: small RNAs with a big role in gene regulation. Nat Rev Genet.

[5] Bushati N, Cohen SM (2007). microRNA functions. Annu Rev Cell Dev Biol.

[6] Lu J, Getz G, Miska EA, Alvarez-Saavedra E, Lamb J, Peck D, Sweet-Cordero A, Ebert BL, Mak RH, Ferrando AA, Downing JR, Jacks T, Horvitz HR (2005). MicroRNA expression profiles classify human cancers. Nature.

[7] Volinia S, Calin GA, Liu CG, Ambs S, Cimmino A, Petrocca F, Visone R, Iorio M, Roldo C, Ferracin M, Prueitt RL, Yanaihara N, Lanza G (2006). A microRNA expression signature of human solid tumors defines cancer gene targets. Proc Natl Acad Sci U S A.

[8] Calin GA, Sevignani C, Dumitru CD, Hyslop T, Noch E, Yendamuri S, Shimizu M, Rattan S, Bullrich F, Negrini M, Croce CM (2004). Human microRNA genes are frequently located at fragile sites and genomic regions involved in cancers. Proc Natl Acad Sci U S A.

[9] He L, Thomson JM, Hemann MT, Hernando-Monge E, Mu D, Goodson S, Powers S, Cordon-Cardo C, Lowe SW, Hannon GJ, Hammond SM (2005). A microRNA polycistron as a potential human oncogene. Nature.

[10] Diosdado B, van de Wiel MA, Terhaar Sive Droste JS, Mongera S, Postma C, Meijerink WJ, Carvalho B, Meijer GA (2009). MiR-17-92 cluster is associated with 13q gain and c-myc expression during colorectal adenoma to adenocarcinoma progression. Br J Cancer.

[11] Chen ZL, Zhao XH, Wang JW, Li BZ, Wang Z, Sun J, Tan FW, Ding DP, Xu XH, Zhou F, Tan XG, Hang J, Shi SS (2011). microRNA-92a promotes lymph node metastasis of human esophageal squamous cell carcinoma via E-cadherin. J Biol Chem.

[12] Ng EK, Chong WW, Jin H, Lam EK, Shin VY, Yu J, Poon TC, Ng SS, Sung JJ (2009). Differential expression of microRNAs in plasma of patients with colorectal cancer: a potential marker for colorectal cancer screening. Gut.

[13] Tanaka M, Oikawa K, Takanashi M, Kudo M, Ohyashiki J, Ohyashiki K, Kuroda M (2009). Down-regulation of miR-92 in human plasma is a novel marker for acute leukemia patients. PLoS One.

[14] Nilsson S, Moller C, Jirstrom K, Lee A, Busch S, Lamb R, Landberg G (2012). Downregulation of miR-92a Is Associated with Aggressive Breast Cancer Features and Increased Tumour Macrophage Infiltration. PLoS One.

[15] Shin VY, Ng EK, Chan VW, Kwong A, Chu KM (2015). A three-miRNA signature as promising non-invasive diagnostic marker for gastric cancer. Mol Cancer.

[16] Yeh TS, Wu CW, Hsu KW, Liao WJ, Yang MC, Li AF, Wang AM, Kuo ML, Chi CW (2009). The activated Notch1 signal pathway is associated with gastric cancer progression through cyclooxygenase-2. Cancer Res.

[17] Ota A, Tagawa H, Karnan S, Tsuzuki S, Karpas A, Kira S, Yoshida Y, Seto M (2004). Identification and characterization of a novel gene, C13orf25, as a target for 13q31-q32 amplification in malignant lymphoma. Cancer Res.

[18] Nishida N, Nagahara M, Sato T, Mimori K, Sudo T, Tanaka F, Shibata K, Ishii H, Sugihara K, Doki Y, Mori M (2012). Microarray Analysis of Colorectal Cancer Stromal Tissue Reveals Upregulation of Two Oncogenic miRNA Clusters. Clin Cancer Res.

[19] Motoyama K, Inoue H, Takatsuno Y, Tanaka F, Mimori K, Uetake H, Sugihara K, Mori M (2009). Over- and under-expressed microRNAs in human colorectal cancer. Int J Oncol.

[20] Shigoka M, Tsuchida A, Matsudo T, Nagakawa Y, Saito H, Suzuki Y, Aoki T, Murakami Y, Toyoda H, Kumada T, Bartenschlager R, Kato N, Ikeda M (2010). Deregulation of miR-92a expression is implicated in hepatocellular carcinoma development. Pathol Int.

[21] Hsu KW, Hsieh RH, Huang KH, Fen-Yau Li A, Chi CW, Wang TY, Tseng MJ, Wu KJ, Yeh TS (2012). Activation of the Notch1/STAT3/Twist signaling axis promotes gastric cancer progression. Carcinogenesis.

[22] Tseng YC, Tsai YH, Tseng MJ, Hsu KW, Yang MC, Huang KH, Li AF, Chi CW, Hsieh RH, Ku HH, Yeh TS (2012). Notch2-induced COX-2 expression enhancing gastric cancer progression. Mol Carcinog.

[23] Sun Y, Gao X, Liu J, Kong QY, Wang XW, Chen XY, Wang Q, Cheng YF, Qu XX, Li H (2011). Differential Notch1 and Notch2 expression and frequent activation of Notch signaling in gastric cancers. Arch Pathol Lab Med.

[24] Shin VY, Jin HC, Ng EK, Cho CH, Leung WK, Sung JJ, Chu KM (2011). 4-(Methylnitrosamino)-1-(3-pyridyl)-1-butanone promoted gastric cancer growth through prostaglandin E receptor (EP2 and EP4) in vivo and in vitro. Cancer Sci.

[25] Shin VY, Jin H, Ng EK, Cheng AS, Chong WW, Wong CY, Leung WK, Sung JJ, Chu KM (2011). NF-kappaB targets miR-16 and miR-21 in gastric cancer: involvement of prostaglandin E receptors. Carcinogenesis.

[26] Xie C, Liang B, Xue M, Lin AS, Loiselle A, Schwarz EM, Guldberg RE, O’Keefe RJ, Zhang X (2009). Rescue of impaired fracture healing in COX-2-/- mice via activation of prostaglandin E2 receptor subtype 4. Am J Pathol.

[27] Hatazawa R, Tanigami M, Izumi N, Kamei K, Tanaka A, Takeuchi K (2007). Prostaglandin E2 stimulates VEGF expression in primary rat gastric fibroblasts through EP4 receptors. Inflammopharmacology.

[28] Iwanaga K, Okada M, Murata T, Hori M, Ozaki H (2012). Prostaglandin E2 promotes wound-induced migration of intestinal subepithelial myofibroblasts via EP2, EP3, and EP4 prostanoid receptor activation. J Pharmacol Exp Ther.

[29] Xin X, Majumder M, Girish GV, Mohindra V, Maruyama T, Lala PK (2012). Targeting COX-2 and EP4 to control tumor growth, angiogenesis, lymphangiogenesis and metastasis to the lungs and lymph nodes in a breast cancer model. Lab Invest.

[30] Shin VY, Siu JM, Cheuk I, Ng EK, Kwong A (2015). Circulating cell-free miRNAs as biomarker for triple-negative breast cancer. Br J Cancer.

[31] Shin VY, Wu WK, Chu KM, Wong HP, Lam EK, Tai EK, Koo MW, Cho CH (2005). Nicotine induces cyclooxygenase-2 and vascular endothelial growth factor receptor-2 in association with tumor-associated invasion and angiogenesis in gastric cancer. Mol Cancer Res.

[32] Faustino-Rocha A, Oliveira PA, Pinho-Oliveira J, Teixeira-Guedes C, Soares-Maia R, da Costa RG, Colaco B, Pires MJ, Colaco J, Ferreira R, Ginja M (2013). Estimation of rat mammary tumor volume using caliper and ultrasonography measurements. Lab Anim (NY).

